# Forest Conversion Drives Divergent Responses in Bird and Mammal Diversity: Stand Structure Matters for Birds, Elevation for Mammals

**DOI:** 10.3390/ani16111725

**Published:** 2026-06-04

**Authors:** Xiangxiang Chen, Tianyu Huang, Ru Li, Rui Yang, Yan He, Shuai Zou, Lixiao Yi, Xiaoyue Lin, Jianping Ying, Jingkai Lai, Yuxin Ye, Sili Peng, Zhiwei Ge

**Affiliations:** 1 Co-Innovation Center for Sustainable Forestry in Southern China, College of Ecology and Environment, Nanjing Forestry University, Nanjing 210037, China; xiangxchen@njfu.edu.cn (X.C.); 15851668808@163.com (T.H.); 13869772367@163.com (R.L.); yangrui97482@njfu.edu.cn (R.Y.); heyan@njfu.edu.cn (Y.H.); zoushuai1618@126.com (S.Z.); pengsili@njfu.edu.cn (S.P.); 2 Longyou County Forestry Technology Extension Station, Quzhou 324403, China; ylx8358@163.com (L.Y.); lyxlyslj@163.com (J.L.); 3 Longyou County Xikou Forestry Farm, Quzhou 324403, China; lxy4989@sohu.com; 4 Longyou County Forest Resources Monitoring Station, Quzhou 324403, China; cinci1983@163.com; 5 Miaoxia Township People’s Government of Longyou County, Quzhou 324000, China; 17858563031@163.com

**Keywords:** infrared-triggered camera, phylogenetic diversity, animal conservation, forest management, altitudinal gradient

## Abstract

Forest conversion from natural forests to secondary forests and plantations has significantly altered wildlife habitats in subtropical regions. However, the drivers of disparities in bird and mammal multidimensional diversity between these forest types remain poorly understood. We analyzed a four-year camera-trapping dataset to compare the taxonomic, functional, and phylogenetic diversity and community structure of birds and mammals. Our results indicated that forest conversion impacts biodiversity differently across taxa. Birds exhibited higher taxonomic and phylogenetic diversity in secondary forests than in plantations. A similar pattern was also evident for birds among different stand types. However, mammals exhibited considerable taxonomic diversity but showed higher phylogenetic diversity and structure in secondary forests. A similar pattern was also evident for mammals among different stand types. Beta diversity revealed significant differences in bird taxonomic composition and mammal phylogenetic composition between secondary and plantation forests. Furthermore, elevation primarily influenced bird taxonomic diversity, phylogenetic diversity and structure in secondary forests, whereas mammal functional diversity, phylogenetic diversity and structure were more sensitive to elevational changes in both secondary and plantation forests. These findings reveal that birds and mammals respond distinctively to forest conversion. We emphasize that management strategies must be group-specific. For birds, we recommend prioritizing the preservation of secondary forests as biodiversity refugia and transforming structurally simplified plantations into complex habitats by retaining legacy trees and native understory vegetation. For mammals, conservation should prioritize landscape-scale connectivity by protecting continuous forest corridors along altitudinal gradients. Practically, this requires restricting further fragmentation of high-altitude habitats and restoring native vegetation in degraded corridors to facilitate dispersal and maintain the phylogenetic integrity of mammal communities.

## Simple Summary

In subtropical regions, natural forests are increasingly being replaced by secondary and plantation forests. However, how these changes affect wildlife remains unclear. We used a four-year camera-trapping dataset to analyze the multidimensional diversity of birds and mammals in secondary and plantation forests in eastern China. Our results elucidated that both birds and mammals exhibited significant differences in their taxonomic, functional, and phylogenetic diversity between the two forest types. Beta diversity analysis showed that taxonomic and phylogenetic composition differed significantly between forest types. Notably, stand types were a stronger driver for bird diversity, while altitude played a more critical role in shaping mammal communities. These findings suggest that forest management strategies must account for both forest type and elevational connectivity to effectively protect montane wildlife.

## 1. Introduction

China’s forest transition reached its critical turning point in the 1980s, with state policies serving as a key driving force [1,2]. This was accompanied by a decrease in natural forest area and an increase in secondary and plantation forest areas [3]. Against this background, understanding the diversity and community assembly of animals in different forest types facilitates a balanced approach to the conservation of forest vegetation and animals and provides a deeper understanding of the historical context of animal community assembly in various habitats [4]. Forest animals play a vital role in maintaining ecosystem functions and services. Specifically, they facilitate forest regeneration by acting as resource linkers (energy flow), genetic linkers (seed propagation and pollination), and process linkers (cross-habitat foragers and prey). The loss or alteration of these roles can significantly hinder the ability of plant communities to adapt to climate change [5,6,7,8]. Birds and mammals, as key species for forest ecological health [7,9], are a powerful social motivator for conservation [10]. However, the fragmentation of habitats has accelerated the extinction of animals in recent decades [11,12]. Furthermore, the transition from natural forests to secondary and plantation forests significantly reduces wildlife diversity, as well as related ecosystem functions and services [13,14]. Given that secondary and plantation forests are becoming the dominant forest types under increasing human interference [15], it is crucial to focus on the dynamics and drivers of bird and mammal diversity within these habitats. Such research is essential for developing forest management strategies that effectively balance biodiversity conservation with anthropogenic land use [16].

While taxonomic diversity provides a baseline for assessing species richness, it often obscures the mechanisms by which species partition resources in response to habitat changes [17,18]. Forest conversion, specifically the transition from secondary forests to simplified plantations, frequently leads to the loss of complex structural niches, necessitating trait-based approaches to understand the resulting impact on community resilience [18,19]. By categorizing species into functional groups [20], we can better analyze how environmental disturbances in managed forests drive the loss of specific functional traits [21,22,23]. Despite growing interest in how habitat fragmentation affects functional diversity [24,25,26], the specific functional consequences of secondary-to-plantation forest conversion remain underexplored. Our study addresses this knowledge gap by determining whether this transition induces a functional bottleneck that disproportionately affects species performing key ecosystem services.

Phylogenetic diversity and community assembly analyses offer a mechanistic lens to identify the processes driving biodiversity in modified landscapes [27,28]. Specifically, community assembly theory posits that closely related species often share similar functional traits and occupy comparable niches [29]. By quantifying phylogenetic structure (NRI, NTI), we can distinguish between community assembly driven by environmental filtering (leading to phylogenetic clustering) and that driven by competitive exclusion (leading to phylogenetic overdispersion) [4]. While previous studies have addressed the broad-scale drivers of phylogenetic diversity [28,30,31], the specific phylogenetic signatures of forest conversion from secondary forests to structurally simplified plantations remain poorly resolved. Our study utilizes these metrics to test whether the transition to plantation forests imposes a stronger environmental filter on evolutionary lineages, thereby providing a deeper understanding of how habitat simplification reshapes the phylogenetic integrity of forest animal communities.

Although numerous studies have investigated animal diversity and community structure across forest types [31,32,33,34], the mechanistic drivers behind diversity disparities in subtropical secondary and plantation forests remain poorly resolved. We hypothesize that habitat simplification in plantations imposes stronger environmental filtering on forest-dependent species, whereas secondary forests maintain higher multidimensional diversity through complex structural niches. Based on this, we address three specific objectives: (1) to evaluate whether bird and mammal communities exhibit significant taxon-specific responses to forest conversion, (2) to assess how stand types influence community changes, predicting that bird communities are primarily structured by local stand complexity given their specialization in forest structure; and (3) to analyze altitudinal variation, anticipating that mammal communities will show a stronger elevational signal due to their more limited dispersal capacity and specific physiological constraints compared to birds.

## 2. Materials and Methods

### 2.1. Study Areas and Animal Monitoring

**Figure 1:**
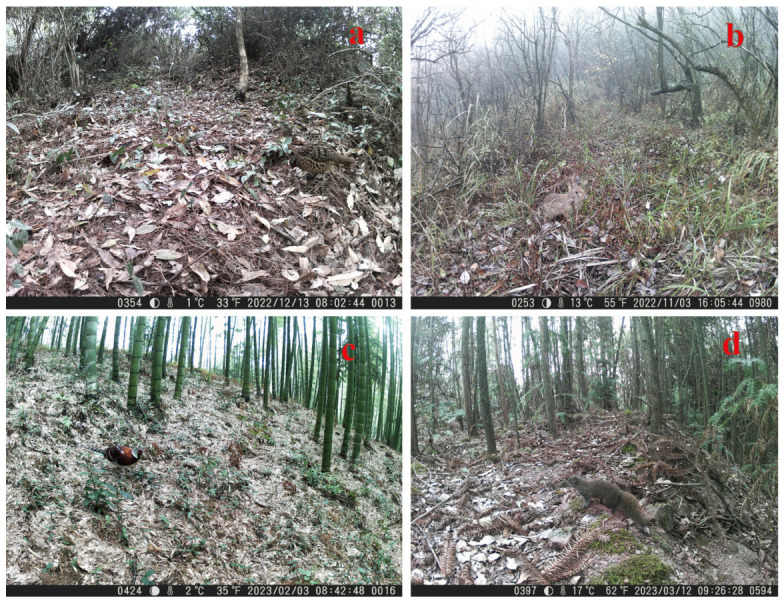
An overview of the four stand types of forest surveyed in Longyou County, Zhejiang Province, showing their differences in vegetation structure. (**a**) Broad-leaved, (**b**) Mountain shrub, (**c**) Moso bamboo, (**d**) *Cunninghamia lanceolata*.

The study site is located in Longyou County, Zhejiang Province, within China’s subtropical region (28°44′–29°17′ N, 119°02′–119°20′ E). The county spans 61.5 km from north to south and 29.4 km from east to west, covering a total area of 1143 km^2^ with elevations ranging from 33 to 1442 m. This county comprises 2 subdistricts and 13 townships. Our study was conducted across 9 of these townships, where cameras were primarily deployed in mountainous areas far from urban centers to minimize urban influence. The region experiences a subtropical monsoon climate characterized by high humidity, abundant rainfall, and distinct wet and dry seasons. The annual mean temperature and annual mean precipitation are 17.1 °C and 1602.6 mm, respectively. The predominant forest stand types in the county are secondary and plantation forests (Figure 1). We defined secondary forests as naturally regenerated stands that have developed through long-term succession following previous disturbances, characterized by diverse native tree species, high structural complexity, and minimal human intervention (typically >20 years without major logging). In contrast, plantation forests were defined as monoculture stands established for timber or bamboo production, characterized by intensive periodic management and simplified species composition. Notably, while management intensity in plantation forests has recently declined due to regional economic shifts, these stands still exhibit more intensive anthropogenic footprints compared to the relatively stable secondary forests. Secondary forest primarily comprises broad-leaved (BR; elevation 68–1222 m; canopy closure ~60%; including *Castanopsis eyrie*, *Schima superba*, *Castanopsis sclerophylla*, *Quercus glauca*, etc.) and mountain shrub (MS; elevation 1030–1368 m; canopy closure ~40%; including *Rhododendron fortunei*, *Lindera reflexa*, *Hydrangea chinensis*, etc.) forests. Plantation forests are dominated by Moso bamboo (MB; elevation 94–968 m; canopy closure ~80% and *Cunninghamia lanceolata* (CL; elevation 87–1255 m; canopy closure ~50%).

We employed 203 infrared-triggered cameras (Model H805, Hong’e Electronics Technology Co., Ltd., in Kunshan, China) that utilized thermal sensing technology to detect animal body temperature and automatically capture high-definition photographs and videos. The infrared camera technology was widely used for surveying medium-to-large mammals, diurnal small mammals, and ground-dwelling birds [35], as it provided repeatable, comparable and sustainable data. In this study, the birds detected by infrared-triggered cameras were predominantly ground-dwelling species. Among these, the ground-dwelling birds mainly included the orders Columbiformes, Galliformes and Passeriformes. These cameras were deployed throughout Longyou County over a four-year period from 2021 to 2024 (Figure S1). Camera deployment covered the entire altitudinal gradients, encompassing nearly all animal habitats [36]. We established 29 camera transects. Each transect was longer than 5 km and equipped with more than five camera traps. In this study, cameras were deployed along elevational gradients from low to high altitudes. The heterogeneity of the mountainous terrain and the method of extracting independent effective photographs also objectively counteracted spatial independence and community similarity. All transects were sampled simultaneously for the collection of both bird and mammal data. A total of 203 cameras were initially deployed; after removing units that failed, were lost, or operated for a significantly shorter period, 187 cameras were finally retained for the subsequent data analysis of this study. The camera parameters and deployment settings followed a standardized protocol [35], and the cameras were deployed at least 200 m apart, considering topographic and vegetation cover characteristics. The cameras were installed on a tree trunk about 0.5 m above the ground, facing parallel to the ground. Data were collected at six-month intervals. Images of the same species captured within 30 min at a single camera station were considered a single independent record [37], minimizing the risk of repeatedly counting the same individual. Species identification primarily followed A Field Guide to the Birds of China [38], A Guide to the Mammals of China [39], and Species 2000 China Node (http://col.especies.cn/). Complete species lists for birds and mammals are provided (Tables S1 and S2).

### 2.2. Data Collection and Analysis

We selected three key functional traits for mammals: body mass, diet (categorized as herbivore, omnivore, or carnivore), and habitat breadth (defined as the number of distinct suitable habitat types inhabited by a species) [40]. For birds, three primary functional traits were collected: body mass, habitat preference, and trophic niche, primarily sourced from the AVONET database [41]. Moreover, strong phylogenetic signals have been detected across these traits, indicating their evolutionary conservatism. These selected traits are widely recognized as standard proxies for assessing functional diversity and have been shown to reflect species’ morphological characteristics, functional roles, and adaptive strategies [31,42,43]. Specifically, these traits were selected to capture functional responses to forest conversion: body mass reflects sensitivity to space constraints and human disturbance, while diet and habitat breadth indicate adaptations to food web simplification and habitat homogenization. In terms of diversity analysis, animal taxonomic diversity was analyzed using the ‘diversity’ function in the *vegan* package (version 2.7-1) within the R environment [44,45]. Taxonomic diversity was evaluated by the four metrics: the Margalef index, Simpson index, Shannon index, and Pielou index. The Margalef index assesses species richness within communities [46]. The Simpson diversity index (Simpson index) quantifies species dominance, with lower indices indicating higher community diversity [47]. The Shannon-Wiener index (Shannon index) provides a comprehensive measure incorporating both species richness and evenness [48]. The Pielou evenness index (Pielou index) evaluates species abundance evenness within communities [44,45]. For functional diversity analysis, we used the *mFD* package (version 1.0.7) in R to calculate three foundational indices: functional evenness, functional richness, and functional divergence (FDiv) [49]. These indices are mutually independent. Functional evenness measures the regularity of abundance distribution in functional space, reflecting the completeness of resource utilization. Functional richness represents the volume of functional space occupied by the community, indicating the breadth of resource utilization. Functional divergence quantifies the divergence in abundance distribution, reflecting the degree of niche differentiation among species within the community.

To calculate phylogenetic diversity and structure, we used updated phylogenetic trees for birds, which are more accurate in terms of branch lengths and species divergences [50]. For mammals, we used the published phylogeny [51,52]. These global phylogenies were pruned to include only species detected in our study using the *phytools* package (version 2.5-2) in R [53]. We calculated three phylogenetic diversity metrics using the *picante* package (version 1.8.2): Faith’s phylogenetic diversity (PD) [54], mean pairwise distance (MPD), and mean nearest taxon distance (MNTD) [4]. The PD represents the sum of phylogenetic branch lengths for all species in a community. MPD is the average phylogenetic distance between all possible species pairs, while MNTD is the average distance between each species and its closest relative [55]. Community assembly was assessed using two parameters: the net relatedness index (NRI) and nearest taxon index (NTI) in the *picante* package using the ‘frequency’ null model, which quantify phylogenetic relatedness of species assemblages [56]. The ‘frequency’ null model was selected because camera-trap data are inherently frequency-based, and the ‘frequency’ model outperformed the default ‘taxa.labels’ model in our previous analyses. The NRI and NTI measure the standardized effect size of MPD and MNTD, respectively [55,57]. NRI reflects overall phylogenetic relatedness, whereas NTI focuses on local phylogenetic relatedness [58]. Phylogenetic clustering is positively correlated with both NRI and NTI [4,59,60]. Values greater than zero indicate phylogenetic clustering, values less than zero suggest phylogenetic overdispersion, and values equal to zero indicate random community structure [61].

To comprehensively assess the impact of forest conversion on animal communities across multiple dimensions, we calculated beta diversity, conducted ordination, and performed statistical tests from taxonomic, functional, and phylogenetic perspectives. Taxonomic beta diversity was analyzed based on Bray–Curtis dissimilarity matrices computed using the ‘vegdist’ function of the *vegan* package, which were subsequently used for non-metric multidimensional scaling (NMDS) ordination and visualization using the ‘metaMDS’ function of the *vegan* package to illustrate community composition differences. Functional beta diversity was analyzed based on Gower distance matrices computed using the daisy function of the *cluster* package (version 2.1.6), with NMDS ordination applied to visualize patterns in functional trait space. Phylogenetic beta diversity was analyzed based on mean phylogenetic distance matrices computed using the ‘comdist’ function of the *picante* package and similarly visualized using NMDS ordination. For each dimension, we performed PERMANOVA tests using the ‘adonis2’ function of the *vegan* package to assess the compositional differences between secondary and plantation forests [62] and conducted PERMDISP tests using the ‘betadisper’ function of the *vegan* package to evaluate the homogeneity of group dispersions [63]. This series of analyses aimed to systematically reveal the patterns of composition and dispersion for animal communities across multiple ecological dimensions in the two forest types.

Finally, to understand the significant differences in animal diversity and community structure metrics between secondary and plantation forests, we performed the Mann–Whitney U test from the *rstatix* package (version 0.7.2) [64]. For comparisons across more than two stand types, the Kruskal–Wallis test was first applied, followed by pairwise post hoc comparisons using Dunn’s test with Bonferroni correction [65]. Furthermore, the ordinary least squares (OLS) regression was used to analyze relationships between altitude and diversity indices [66], as polynomial models did not significantly improve explanatory power, and OLS offers better parsimony and interpretability for the monotonic elevational pattern. Before model fitting, all diversity indices were visually inspected for normality. Model residuals were checked for normality and homoscedasticity to ensure OLS assumptions were met. All statistical analyses were conducted in R software (version 4.4.0) [67].

## 3. Results

### 3.1. Bird and Mammal Species with Co-Occurrence Records

A total of 44,587 independent records were obtained from 187 infrared cameras (Table S3). For birds, 170 cameras recorded 72 species (8 orders, 25 families), generating 17,088 independent records and averaging 114 detections per camera. Among them, 70 bird species were recorded in secondary forests, 57 species in plantations, and 55 species were shared. The total number of independent records for birds was 13,208 in secondary forests and 3880 in plantations. For mammals, 183 cameras documented 19 species (7 orders, 12 families), with 27,499 independent records and an average of 170 detections per camera. Among them, 18 mammal species were recorded in secondary forests, 17 species in plantations, and 16 species were shared. The total number of independent records for mammals was 17,748 in secondary forests and 9751 in plantations. Animal independent record counts were significantly higher in secondary forest than in plantations (bird: W = 5820, *p* < 0.001; mammal: W = 63,273, *p* < 0.001). The mean independent records per camera were significantly higher in secondary forests (bird: 167 ± 19; mammal: 225 ± 21) than in plantations (bird: 43 ± 5; mammal: 94 ± 11).

**Figure 2:**
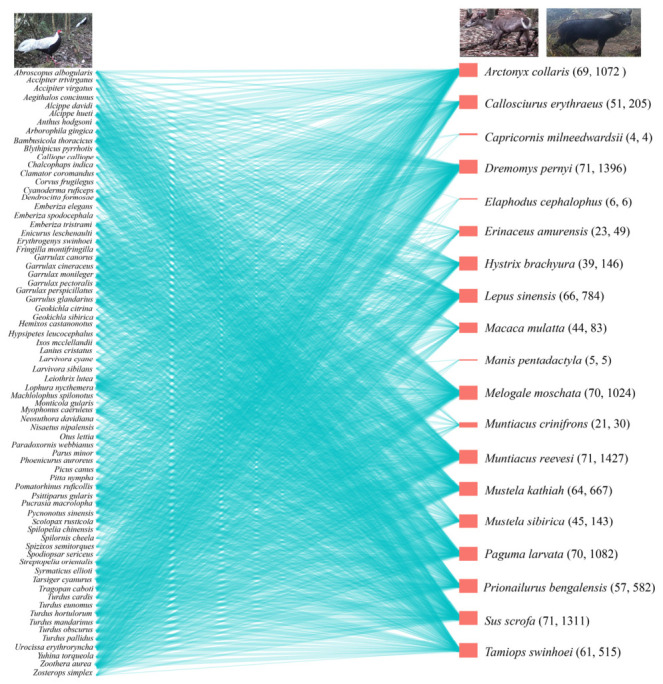
Network analysis of co-occurrence and encounter frequencies between bird and mammal species captured by infrared-triggered cameras. The left nodes represent bird species (*n* = 72), and the right nodes represent mammal species (*n* = 19). The presence of a blue connecting line indicates a co-occurrence between a specific bird and mammal species within the same camera site, with the total number of lines linked to a mammal reflecting the richness of its co-occurring bird species. For each mammal, the values in the red parentheses (X, Y) on the right denote the richness of co-occurring bird species (X: the height of the red square) and their total number of independent effective records (Y), respectively.

In this study, four cameras detected only birds, recording 12 independent records of 9 bird species. Conversely, seventeen cameras detected only mammals, yielding 701 independent records of 11 mammal species. The remaining 166 cameras recorded both taxa, collectively capturing all 72 bird species and 19 mammal species, yielding 43,874 effective records. Among the species with co-occurrence records (Figure 2), detection frequencies varied substantially between birds and mammals. Six mammal species exhibited low detection frequencies (fewer than 100 records): *Capricornis milneedwardsii* (Bovidae), *Manis pentadactyla* (Manidae), *Elaphodus cephalophus* (Cervidae), *Muntiacus crinifrons* (Cervidae), *Erinaceus amurensis* (Erinaceidae), and *Macaca mulatta* (Cercopithecidae). In contrast, six mammal species were detected more than 1000 times: *Melogale moschata* (Mustelidae), *Arctonyx collaris* (Mustelidae), *Paguma larvata* (Prionodontidae), *Sus scrofa* (Suidae), *Dremomys pernyi* (Sciuridae), and *Muntiacus reevesi* (Cervidae). Infrared-triggered camera monitoring revealed that independent records of birds and mammals differed within the same habitat. Specifically, based on the Mann–Whitney U test, both bird richness and detection frequency tended to be lower in habitats with endangered mammals but higher in areas with common mammals (richness: *W* = 36, *p* < 0.01; frequency: *W* = 36, *p* < 0.01).

### 3.2. Bird and Mammal Diversity and Community Structure Between Secondary and Plantation Forests

**Figure 3:**
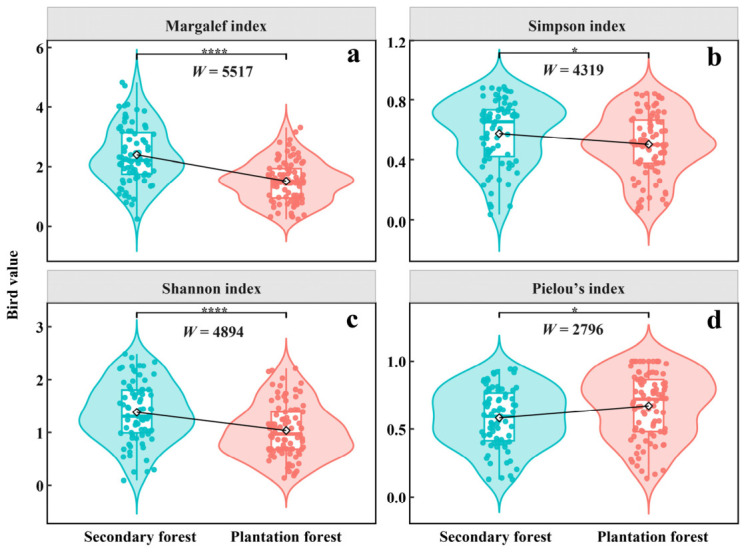
The differences in bird taxonomic diversity between secondary (*n* = 79) and plantation (*n* = 91) forests based on the Mann–Whitney U test. (**a**) Margalef index; (**b**) Simpson index; (**c**) Shannon index; (**d**) Pielou’s index. The diamond symbols represent the mean value of the specific metric. * *p* < 0.05, **** *p* < 0.0001.

Multidimensional diversity and community structure varied significantly between forest types, with distinct response patterns observed for birds and mammals (Figure 3 and Figure S2). For birds, the secondary forests (SF) supported significantly higher taxonomic (Margalef, Simpson, and Shannon) and phylogenetic diversity (Faith’s PD) than plantation forests (PF). However, PF exhibited higher taxonomic (Pielou), functional evenness (FEve) and phylogenetic diversity (MPD, MNTD). Other functional (FRic, FDiv) and community structure (NRI, NTI) indices showed no significant differences between forest types. For mammals, taxonomic diversity was largely comparable between SF and PF, with the exception of the Pielou index, which was higher in PF. In contrast to birds, mammal functional richness (FRic), phylogenetic diversity (Faith’s PD) and phylogenetic clustering (NRI and NTI) were significantly higher in SF, while MPD and MNTD were significantly lower in PF. These results indicated that SF harbored communities composed of more closely related lineages, while PF harbored communities composed of more distantly related lineages.

**Figure 4:**
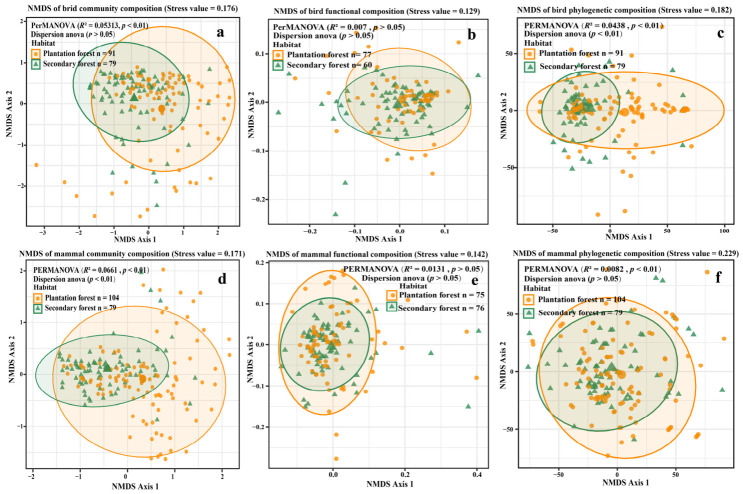
(**a**) Taxonomic composition ordination of birds; (**b**) Functional composition ordination of birds; (**c**) Phylogenetic composition ordination of birds; (**d**) Taxonomic composition ordination of mammals; (**e**) Functional composition ordination of mammals; (**f**) Phylogenetic composition ordination of mammals. The PERMANOVA and PERMDISP results indicate differences in community, functional, and phylogenetic composition and dispersion. Ellipses represent 95% confidence intervals for each group.

The beta diversity revealed distinct patterns of compositional shifts for birds and mammals between secondary and plantation forests (Figure 4). For birds, PERMANOVA showed a significant location effect in community composition, while the non-significant PERMDISP confirmed that this difference reflected genuine compositional shifts rather than variations in dispersion (Figure 4a). However, no significant differences were found in bird functional composition (Figure 4b). Interestingly, bird phylogenetic composition exhibited significant differences in both location and dispersion (Figure 4c). For mammals, both community composition and its dispersion differed significantly between forest types (Figure 4d). Similar to birds, mammal functional composition remained comparable across habitats (Figure 4e). Notably, mammal phylogenetic composition showed a pure location effect, with significant PERMANOVA results but stable dispersion (Figure 4f).

### 3.3. Bird and Mammal Diversity and Community Structure Among Different Stand Types

Distinct patterns in diversity characteristics were observed for birds and mammals across forest types (Table 1). For bird communities, stand types within secondary forests supported significantly higher taxonomic (Margalef, Simpson, Shannon) and phylogenetic (Faith’s PD) diversity than those within plantation forests but were lower in the taxonomic (Pielou), functional (FEve), and phylogenetic (MPD, MNTD) diversity indices. Notably, bird community structure (NRI and NTI) remained comparable among different stands. In contrast, stand types within secondary forests were characterized by significantly higher mammal Faith’s PD and NTI but significantly lower Pielou, MPD, and MNTD indices compared to those within plantations.

### 3.4. The Relationship Between Altitude and Bird and Mammal Diversity and Community Structure

**Figure 5:**
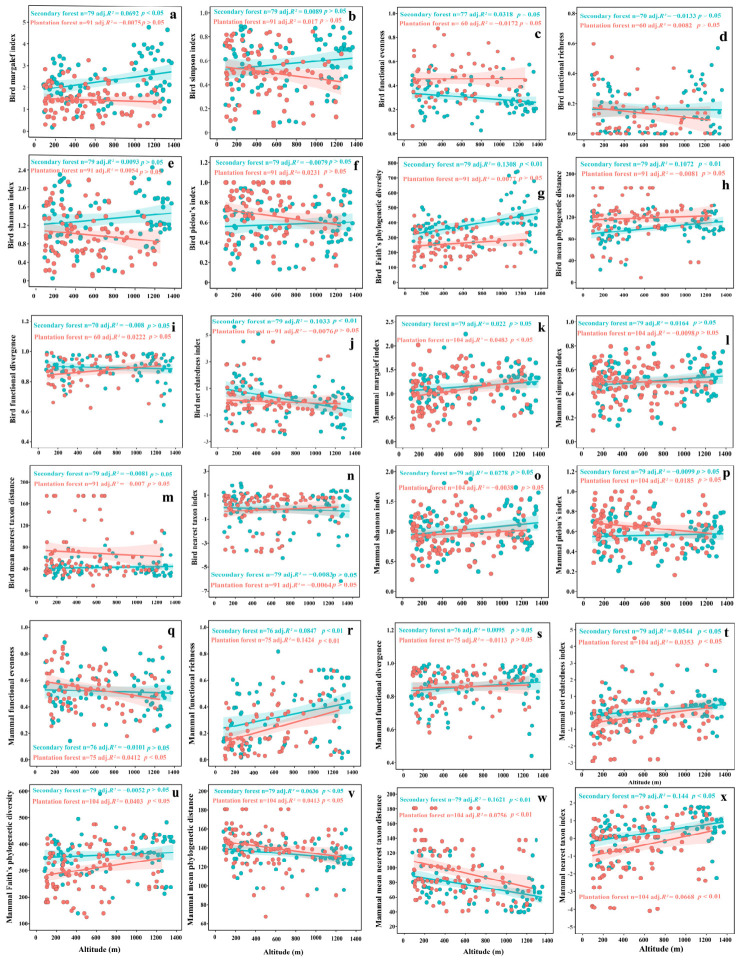
Correlation between altitude and diversity and community structure of birds and mammals in secondary and plantation forests by an ordinary least squares regression analysis. For bird, taxonomic diversity (**a**,**b**,**e**,**f**); functional diversity (**c**,**d**,**i**); phylogenetic diversity (**g**,**h**,**m**); community structure indices (**j**,**n**). For Mammal, taxonomic diversity (**k**,**l**,**o**,**p**); functional di-versity (**q**–**s**); phylogenetic diversity (**u**–**w**); community structure indices (**t**,**x**). For birds, the group samples of different forests are from 60 to 91. For mammals, the group samples of different forests are from 75 to 104. The shaded areas represent the 95% confidence intervals.

In bird taxonomic diversity, the altitude only showed a significant positive correlation with the Margalef index in secondary forests (adj. *R*^2^ = 0.0692, *p* < 0.05) (Figure 5a). In bird phylogenetic diversity, there was significant positive correlation between altitude and Faith’s PD (adj. *R*^2^ = 0.1308, *p* < 0.01) as well as MPD (adj. *R*^2^ = 0.1072, *p* < 0.01) in the secondary forests (Figure 5g,h). In bird community structure, the NRI only had a significant negative correlation with altitude in secondary forests (adj. *R*^2^ = 0.1033, *p* < 0.01) (Figure 5j). For mammals, the altitude only showed a significant positive correlation with the Margalef index (adj. *R*^2^ = 0.0483, *p* < 0.05) in plantation forests (Figure 5k). In mammal functional diversity, the FRic showed a significant positive correlation with altitude in secondary and plantation (adj. *R*^2^ = 0.0847, *p* < 0.01) forests (Figure 5r), while the FEve (adj. *R*^2^ = 0.1424, *p* < 0.01) exhibited a significant negative correlation with altitude in plantation forests (Figure 5q). In mammal phylogenetic diversity (Figure 5u–w), the altitude had a negative correlation with MPD (adj. *R*^2^ = 0.0636, *p* < 0.05) and MNTD (adj. *R*^2^ = 0.1621, *p* < 0.05) within secondary forests. However, the altitude exhibited significant positive correlation with Faith’s PD (adj. *R*^2^ = 0.0403, *p* < 0.05) and negative correlation with MPD (adj. *R*^2^ = 0.0413, *p* < 0.05) and MNTD (adj. *R*^2^ = 0.0756, *p* < 0.01) in plantation forests. All correlations between altitude and mammal community structure (NRI, NTI) were significantly positive in both secondary and plantation forests (Figure 5t,x). Altitude showed no significant correlations with the other diversity and community structure indices of birds and mammals (Figure 5).

## 4. Discussion

### 4.1. Effects of Forest Types on Animal Diversity and Community Structure

Generally, biodiversity is highest in natural forests, followed by secondary and plantation forests [68]. This pattern may likely stem from the reduction in stand structural complexity and resource availability caused by deforestation [69]. Previous studies have shown that primary forests tend to harbor higher bird species than secondary or plantation forests, although these differences are not always statistically significant [31]. Interestingly, our research found that both bird richness and detection frequency tended to be lower in habitats with endangered mammals but higher in areas with common mammals. This pattern is largely driven by differences in habitat quality, as regions characterized by intense mammal activity typically feature higher primary productivity. Such high environmental heterogeneity naturally sustains both large mammal populations and diverse bird communities [70]. In addition, the frequent behaviors of these mammals may generate favorable microenvironments, which subsequently enhances the species diversity of sympatric birds [71]. For instance, acting as ecosystem engineers, wild boars facilitate resource availability through their rooting behavior. Their disturbance uncovers subterranean flora and fauna, and creates temporal pools, thereby improving food and water access for co-occurrence species [72,73,74]. In bird taxonomic diversity, our results revealed that secondary forests supported higher species richness and a much larger number of bird records. This indicates that, compared to plantations, secondary forests with higher habitat heterogeneity can indeed support more species, as our results found that the Margalef, Simpson and Shannon indices were significantly higher in secondary forests than in plantations. Crucially, the taxonomic beta diversity analysis for birds indicates that the observed differences are driven by a consistent compositional shift rather than variations in community dispersion. This shift in community identity is likely due to the higher habitat heterogeneity and resource carrying capacity of secondary forests, which sustain diverse ecological niches for specialized species [75,76,77]. Interestingly, plantations exhibited significantly higher taxonomic (Pielou) and functional evenness (FEve) than secondary forests. In secondary forests, high habitat heterogeneity and abundant resources may allow certain generalist or dominant species to persist in extremely high abundances, which consequently lowers overall community evenness. Conversely, the simplified vegetation structure and associated resource limitations in plantations may act as environmental filters, preventing any single dominant species from achieving excessive abundance [70]. Furthermore, bird functional composition remained stable across both habitats. The significantly higher FEve in plantations suggests that forest conversion did not alter the types of functional roles present. Rather, it constrained the “richness structure” through resource limitation [49]. This limitation likely forced a more regular occupancy of the available functional space in plantations. In contrast, the lower FEve in secondary forests may reflect higher niche packing and functional clustering among coexisting species. Collectively, these findings suggest that resource limitation in simplified habitats not only constrains overall bird abundance but also facilitates a more even distribution of functional roles among ground-dwelling birds captured by infrared-triggered cameras. Regarding phylogenetic diversity, secondary forests exhibited significantly higher Faith’s PD alongside significantly lower MPD and MNTD. This pattern, coupled with significant shifts in both phylogenetic location and dispersion, demonstrates that secondary forests and plantations harbor fundamentally different evolutionary lineages. The high habitat heterogeneity in secondary forests facilitates the coexistence of a larger number of closely related species, suggesting that these complex habitats act as evolutionary reservoirs that support specific, clustered lineages that are largely absent or restructured in simplified plantation systems.

For mammals, the research found the number of independent records was higher in secondary forests than in plantations, suggesting that structurally complex secondary forests support higher mammal densities through greater environmental heterogeneity [57,70]. Moreover, mammal species richness is inherently low at the regional scale. For instance, only 14 species were recorded over one year of camera trapping in nearby Changshan County [78] and 24 species in a regional nature reserve [79]. The significantly higher mammal Pielou index in plantations may be attributed to simplified habitat structures and limited resources, which likely suppress the dominance of highly competitive species [49]. Crucially, the taxonomic beta diversity revealed significant shifts in both community composition and dispersion, underscoring that forest conversion fundamentally altered not only the identity of mammal species but also the internal variability of their communities. In mammal taxonomic diversity, the mammal functional richness (FRic) was significantly higher in secondary forests than in plantations. However, functional composition remained comparable across habitats, suggesting a degree of functional redundancy where core ecological roles are maintained despite the shrinking functional volume in plantations. In mammal phylogenetic diversity, secondary forests supported significantly higher overall phylogenetic diversity (Faith’s PD) than plantation forests. Furthermore, secondary forests exhibited significantly lower mean pairwise distance (MPD) and mean nearest taxon distance (MNTD) than plantations. This suggests that, throughout the process of forest succession, the high habitat heterogeneity of secondary forests promotes the coexistence of diverse species by providing more expanded niche space [80], thereby accumulating a greater total evolutionary history. Specifically, the complex microhabitat structures in secondary forests may facilitate the co-occurrence of specific clades with similar habitat preferences or adaptive advantages (such as certain specialists among rodents or small carnivores). In contrast, the lower habitat heterogeneity in plantations, coupled with resource scarcity and intensified interspecific competition driven by anthropogenic disturbances, may favor the coexistence of more distantly related mammal lineages [81]. Notably, the restricted distribution of two threatened large herbivores (*Muntiacus crinifrons*, *Capricornis milneedwardsii*) exclusively within secondary forests underscores the irreplaceable conservation value of these habitats. This further emphasizes the critical role of secondary forests in sustaining large ungulate populations and protecting evolutionarily distinct lineages. Finally, the results revealed a significant shift in the phylogenetic structure of mammal communities during the transition from secondary forests to plantations. Mammals shifted from phylogenetic clustering in secondary forests to overdispersion in plantations. This suggests that the high structural complexity of secondary forests provides diverse and specialized microhabitats, facilitating the coexistence of closely related taxa within distinct niches. Conversely, the simplified environment of plantations likely limits these microhabitat refugia, potentially intensifying competitive exclusion among similar species [81]. Alternatively, this overdispersed pattern could also be attributed to environmental filtering, where the restricted resources in plantations selectively favor a small number of distantly related lineages that possess the specific traits required to persist in simplified habitats.

### 4.2. Effects of Different Stand Types on Animal Diversity and Community Structure

Habitat constitutes the spatial matrix for the life processes of individuals, populations, and communities. Animal habitat selection is non-random and characterized by inherent regularities [82]. This preference for specific habitat types directly influences survival and reproductive success [83,84]. The habitat heterogeneity within different forest types has an impact on animal diversity. For instance, *Lophura nycthemera* avoids coniferous forests and shrublands, favoring humid broadleaf forests with high canopy cover [85]. This selection likely facilitates concealment, as conspicuous male plumage contrasts sharply with the environment [86]. Among mammals, the abundance of the primarily herbivorous wild boar varies significantly across different forest types [87,88]. Our results revealed that bird taxonomic diversity (Margalef, Simpson, Shannon and Pielou indices), functional diversity (FEve) and phylogenetic diversity (Faith’s PD, MPD and MNTD) exhibited significant differences among stand types in secondary and plantation forests. In contrast, mammals showed significant differences only in the Pielou index, Faith’s PD, MPD, MNTD and NTI. These findings indicate that variations in stand types have a more pronounced impact on bird diversity indices compared to those of mammals. One potential reason for this divergence is the difference in mobility between the two taxa. Compared to terrestrial mammals, which may be more constrained by topographic barriers, birds generally possess higher dispersal capacity. Although we did not track individual movements directly, the observed community shifts are consistent with our previous findings in this region, which documented significant seasonal altitudinal migrations for both birds and mammals [89]. This suggests that the high mobility of birds may allow for more flexible habitat utilization at a landscape scale, potentially enabling them to aggregate in secondary forests that offer more abundant resources. In contrast, the more localized responses of mammals likely reflect their higher sensitivity to local habitat quality and lower capacity to bypass fragmented plantation patches. Such active migration amplifies the differences in bird communities between secondary and plantation forests, leading to significant divergence across various diversity indices. Regarding resource utilization, secondary forests provide larger ecological niche spaces and shelter for birds with diverse functional traits due to their complex vertical structure and extensive canopy cover [90,91,92,93]. Furthermore, compared to the structurally simple plantations, secondary forests feature denser understory shrubs and herbs. Variations in canopy cover, tree diameter, height, spacing, species diversity, stand biomass, understory vegetation, and deadwood may specifically enhance the habitat preferences of frugivorous or insectivorous birds [31,80]. Consequently, secondary forests with high habitat heterogeneity harbor more specialist species and higher species richness than plantations [75,94], rendering bird diversity levels more sensitive to the stand structural differences between forest types.

### 4.3. Relationships Between Altitude and Animal Diversity and Community Structure

Exploring altitudinal changes offers a unique opportunity to uncover biodiversity patterns and community dynamics [36]. A higher elevational range is often associated with higher environmental heterogeneity. This can provide more ecological niches, refuges and opportunities for species isolation and speciation, thereby enabling more species to coexist [34,70,95]. For instance, the elevation was positively associated with insectivorous bird richness and abundance [96]. The higher elevation range had the higher mammal richness [30]. Our research found that mammal diversity indices exhibited significant correlations with altitude across both forest types, whereas bird diversity showed more limited elevational responses. This indicates that altitudinal changes have a closer relationship with mammals than with birds. While our current data do not allow us to isolate the specific drivers of this divergence, our previous research in this study area has demonstrated that plant diversity and seasonal altitudinal migrations significantly influence mammal community dynamics [89]. Building on these prior findings, the observed patterns may be attributed to the following reasons. Birds with high dispersal ability often transcend elevational and geographical limitations, whereas mammals have limited dispersal capacity and are easily constrained by topographic barriers. In terms of resource utilization, birds can exploit diverse habitats provided by complex forest vertical structure, which may benefit species through greater buffering against altitudinal changes [91,92]. In contrast, mammals rely more on understory space, soil layers, and ground litter [97]. Consequently, elevation-induced fluctuations in understory vegetation cover and microclimate exert a more direct and decisive impact on the food availability and refuge for mammals. Finally, intense anthropogenic disturbances at lower elevations impose greater survival pressure on larger mammals than on birds [98]. Future research utilizing tracking technology or controlled experimental designs is necessary to disentangle the relative importance of these potential ecological drivers. Notably, in both secondary forests and plantations, mammal phylogenetic diversity indices (MPD and MNTD) were significantly negatively correlated with altitude, while community assembly indices (NRI and NTI) increased significantly with elevation. This trend indicates that mammal communities tend toward phylogenetic clustering at higher altitudes, providing strong evidence that environmental filtering plays a dominant role in shaping mammal communities in subtropical high-altitude mountains [4,59,99].

## 5. Conclusions

This study showed that under forest conversion, stand type and altitude significantly shaped bird and mammal diversity. Stand type affected birds more, while altitude affected mammals more. Beyond numerical advantage, the two forest types differed distinctly across taxonomic, functional, and phylogenetic diversity. In addition, beta diversity revealed significant shifts in bird taxonomic composition and mammal phylogenetic composition resulting from forest conversion. To enhance protection, management should move beyond simple coverage. Plantations should prioritize increasing tree diversity and retaining snags to support birds, while establishing forested strips as altitudinal corridors to maintain mammal connectivity across fragmented landscapes. Several limitations require acknowledgment, such as bird data being largely restricted to ground-dwelling birds, spatial autocorrelation not being controlled, inability to distinguish among alternative mechanisms, a single-region focus limiting generalizability, and temporal variation not being fully addressed. Furthermore, unmeasured factors, including human activity intensity, forest age since conversion, understory vegetation density, and food resource availability, may have contributed to unexplained variance. Future research should incorporate a broader range of environmental variables to better disentangle the drivers of animal diversity.

## Figures and Tables

**Table 1:** Differences in bird and mammal diversity and community structure among different stand types based on the Kruskal–Wallis test. For birds, the group samples of different forests are from 21 to 58; For mammals, the group samples of different forests are from 21 to 61. Bold font indicates significant differences among stand types. * *p* < 0.05, ** *p* < 0.01, *** *p* < 0.001, **** *p* < 0.0001.

Category	Index	BR vs. MB	BR vs. CL	MS vs. MB	MS vs. CL
Taxonomic diversity	Margalef (Bird)	BR > MB **** (*Z* = 4.42)	BR > CL *** (*Z* = 4.27)	MS > MB *** (*Z* = 4.18)	MS > CL *** (*Z* = 4.12)
	Margalef (Mammal)	*Z* = −1.49, *p* = 0.818	*Z* = −0.415, *p* = 1	*Z* = −1.57, *p* = 0.7	*Z* = −0.779, *p* = 1
	Simpson (Bird)	*Z* = −0.534, *p* = 1	*Z* = −1.84, *p* = 0.395	*Z* = −2.2, *p* = 0.166	MS > CL * (*Z* = 3.14)
	Simpson (Mammal)	*Z* = −0.671, *p* = 1	*Z* = 0.998, *p* = 1	*Z* = −1.77, *p* = 0.46	*Z* = −0.466, *p* = 1
	Shannon (Bird)	*Z* = −2.36, *p* = 0.11	BR > CL * (*Z* = 2.93)	MS > MB ** (*Z* = 3.28)	MS > CL ** (*Z* = 3.71)
	Shannon (Mammal)	Z = −1.72, *p* = 0.509	*Z* = 0.129, *p* = 1	*Z* = −2.33, *p* = 0.118	*Z* = −0.931, *p* = 1
	Pielou (Bird)	BR < MB ** (*Z* = 3.36)	*Z* = 1.34, *p* = 1	*Z* = 1.05, *p* = 1	*Z* = −0.362, *p* = 1
	Pielou (Mammal)	BR < MB * (*Z* = 2.83)	*Z* = 2.01, *p* = 0.264	*Z* = 1.35, *p* = 1	*Z* = 0.857, *p* = 1
Functional diversity	FEve (Bird)	BR < MB **** (*Z* = 4.32)	BR < CL *** (*Z* = 4.09)	MS < MB *** (*Z* = 3.98)	MS < CL *** (*Z* = 3.83)
	FEve (Mammal)	*Z* = 0.099, *p* = 1	*Z* = 0.97, *p* = 1	*Z* = 0.57, *p* = 1	*Z* = 1.23, *p* = 1
	FRic (Bird)	*Z* = −1.21, *p*= 1	*Z* = −0.257, *p* =1	*Z* = −0.939, *p* = 1	*Z* = −0.189, *p* = 1
	FRic (Mammal)	*Z* = −1.6, *p* = 0.659	*Z* = −2.53, *p* = 0.069	*Z* = −1.9, *p* = 0.346	*Z* = −2.62, *p* = 0.053
	FDiV (Bird)	*Z* = −2, *p* = 0.276	*Z* = −0.884, *p* = 1	*Z* = −0.692, *p* = 1	*Z* = 0.15, *p* = 1
	FDiV (Mammal)	*Z* = −0.439, *p* = 1	*Z* = −0.334, *p* = 1	*Z* = −0.983, *p* = 1	*Z* = −0.893, *p* = 1
Phylogenetic diversity	Faith’s PD (Bird)	BR > MB **** (Z = 5.74)	BR > CL **** (*Z* = 4.40)	MS > MB **** (*Z* = 4.91)	MS > CL *** (*Z* = 3.99)
	Faith’s PD (Mammal)	BR > MB ** (Z = 3.65)	Z = −2.51, *p* = 0.073	MS > MB ** (*Z* = 3.38)	*Z* = −2.59, *p* = 0.057
	MPD (Bird)	BR < MB *** (*Z* = 4.2)	BR < CL ** (Z = 3.28)	*Z* = 2.35, *p* = 0.114	*Z* = 1.77, *p* = 0.464
	MPD (Mammal)	*Z* = 2.17, *p* = 0.18	*Z* = 2.31, *p*= 0.127	*Z* = 2.57, *p* = 0.061	MS < CL * (Z = 2.69)
	MNTD (Bird)	BR < MB ** (*Z* = 3.27)	BR < CL * (Z = 2.88)	MS < MB * (*Z* = 2.67)	Z = 2.44, *p* = 0.088
	MNTD (Mammal)	BR < MB *** (*Z* = 3.83)	*Z* = 2.04, *p* = 0.25	MS < MB *** (*Z* = 3.88)	Z = 2.59, *p* = 0.058
Community Structure	NRI (Bird)	*Z* = −1.19, *p* =1	*Z* = −0.371, *p* = 1	Z = 0.009, *p* = 1	Z = 0.572, *p* = 1
	NRI (Mammal)	*Z* = −2.14, *p* = 0.196	Z = −2.33, *p* = 0.119	Z = −2.34, *p* = 0.116	Z = −2.51, *p* = 0.072
	NTI (Bird)	*Z* = −0.141, *p* = 1	*Z* = 0.287, *p* = 1	Z = −0.592, *p* = 1	Z = −0.25, *p* = 1
	NTI (Mammal)	BR > MB * (*Z* = 3.73)	*Z* = −2.07, *p* = 0.23	MS > MB * (*Z* = 3.65)	Z = −2.47, *p* = 0.082

## Data Availability

The datasets analyzed during the current study are available from the corresponding author on reasonable request.

## Supplementary Materials

The following supporting information can be downloaded at: https://www.mdpi.com/article/10.3390/ani16111725/s1, Table S1. Bird species information at sampling sites monitored by infrared-triggered camera. Table S2. Mammalian species information at sampling sites monitored by infrared-triggered camera. Table S3. Number of bird and mammal studies under different stand types. Figure S1. Habitat information of birds and mammals. Distribution map of 203 infrared-triggered camera sites in Longyou County. yellow circles represent camera sites. Figure S2. Bird and mammal diversity and community structure between secondary and plantation forests. The differences of bird and mammal diversity and community structure between secondary and plantation forests based on Mann-Whitney U test. For birds, the group samples of different forests are from 60 to 91; For mammals, the group samples of different forests are from 75 to 104. The diamond symbols represent the mean value of the specific metric. ** *p* < 0.01, *** *p* < 0.001,**** *p* < 0.0001.
