# Supplementary material for: Forest Conversion Drives Divergent Responses in Bird and Mammal Diversity: Stand Structure Matters for Birds, Elevation for Mammals
Source: Animals (Basel). 2026 Jun 4;16(11):1725. doi: 10.3390/ani16111725 (PMC13255728; doi:10.3390/ani16111725)
Supplement: Supplementary file 1 [file animals-16-01725-s001.zip › animals-4238037-supplementary.pdf]

Forest conversion drives divergent responses in bird and mammal diversity: stand structure matters for birds, elevation for mammals

Supplementary materials

Table S1. Bird species information at sampling sites monitored by infrared-triggered camera

Order	Family	Genus	Species
Accipitriformes	Accipitridae	*Accipiter*	*Accipiter trivirgatus*
Accipitriformes	Accipitridae	*Accipiter*	*Accipiter virgatus*
Accipitriformes	Accipitridae	*Nisaetus*	*Nisaetus nipalensis*
Accipitriformes	Accipitridae	*pilornis*	*Spilornis cheela*
Charadriiformes	Scolopacidae	*Scolopax*	*Scolopax rusticola*
Columbiformes	Columbidae	*Chalcophaps*	*Chalcophaps indica*
Columbiformes	Columbidae	*Spilopelia*	*Spilopelia chinensis*
Columbiformes	Columbidae	*Streptopelia*	*Streptopelia orientalis*
Cuculiformes	Cuculidae	*Clamator*	*Clamator coromandus*
Galliformes	Phasianidae	*Arborophila*	*Arborophila gingica*
Galliformes	Phasianidae	*Bambusicola*	*Bambusicola thoracicus*
Galliformes	Phasianidae	*Lophura*	*Lophura nycthemera*
Galliformes	Phasianidae	*Pucrasia*	*Pucrasia macrolopha*
Galliformes	Phasianidae	*Syrmaticus*	*Syrmaticus ellioti*
Galliformes	Phasianidae	*Tragopan*	*Tragopan caboti*
Passeriformes	Aegithalidae	*Aegithalos*	*Aegithalos concinnus*
Passeriformes	Alcippeidae	*Alcippe*	*Alcippe davidi*
Passeriformes	Alcippeidae	*Alcippe*	*Alcippe hueti*
Passeriformes	Corvidae	*Corvus*	*Corvus frugilegus*
Passeriformes	Corvidae	*Dendrocitta*	*Dendrocitta formosae*
Passeriformes	Corvidae	*Garrulus*	*Garrulus glandarius*
Passeriformes	Corvidae	*Urocissa*	*Urocissa erythroryncha*
Passeriformes	Emberizidae	*Emberiza*	*Emberiza elegans*
Passeriformes	Emberizidae	*Emberiza*	*Emberiza spodocephala*
Passeriformes	Emberizidae	*Emberiza*	*Emberiza tristrami*
Passeriformes	Fringillidae	*Fringilla*	*Fringilla montifringilla*
Passeriformes	Laniidae	*Lanius*	*Lanius cristatus*
Passeriformes	Leiothrichidae	*Garrulax*	*Garrulax canorus*
Passeriformes	Leiothrichidae	*Garrulax*	*Garrulax monileger*
Passeriformes	Leiothrichidae	*Ianthocincla*	*Ianthocincla cineracea*
Passeriformes	Leiothrichidae	*Leiothrix*	*Leiothrix lutea*
Passeriformes	Leiothrichidae	*Pterorhinus*	*Pterorhinus pectoralis*
Passeriformes	Leiothrichidae	*Pterorhinus*	*Pterorhinus perspicillatus*

Order	Family	Genus	Species
Passeriformes	Motacillidae	*Anthus*	*Anthus hodgsoni*
Passeriformes	Muscicapidae	*Calliope*	*Calliope calliope*
Passeriformes	Muscicapidae	*Enicurus*	*Enicurus leschenaulti*
Passeriformes	Muscicapidae	*Larvivora*	*Larvivora cyane*
Passeriformes	Muscicapidae	*Larvivora*	*Larvivora sibilans*
Passeriformes	Muscicapidae	*Monticola*	*Monticola gularis*
Passeriformes	Muscicapidae	*Myophonus*	*Myophonus caeruleus*
Passeriformes	Muscicapidae	*Phoenicurus*	*Phoenicurus auroreus*
Passeriformes	Muscicapidae	*Tarsiger*	*Tarsiger cyanurus*
Passeriformes	Paradoxornithidae	*Neosuthora*	*Neosuthora davidiana*
Passeriformes	Paradoxornithidae	*Psittiparus*	*Psittiparus gularis*
Passeriformes	Paradoxornithidae	*Sinosuthora*	*Sinosuthora webbiana*
Passeriformes	Paridae	*Machlolophus*	*Machlolophus spilonotus*
Passeriformes	Paridae	*Parus*	*Parus minor*
Passeriformes	Pittidae	*Pitta*	*Pitta nympha*
Passeriformes	Pycnonotidae	*Hemixos*	*Hemixos castanonotus*
Passeriformes	Pycnonotidae	*Hypsipetes*	*Hypsipetes leucocephalus*
Passeriformes	Pycnonotidae	*Ixos*	*Ixos mcclellandii*
Passeriformes	Pycnonotidae	*Pycnonotus*	*Pycnonotus sinensis*
Passeriformes	Pycnonotidae	*Spizixos*	*Spizixos semitorques*
Passeriformes	Scotocercidae	*Abroscopus*	*Abroscopus albogularis*
Passeriformes	Sturnidae	*Spodiopsar*	*Spodiopsar sericeus*
Passeriformes	Timaliidae	*Cyanoderma*	*Cyanoderma ruficeps*
Passeriformes	Timaliidae	*Erythrogenys*	*Erythrogenys swinhoei*
Passeriformes	Timaliidae	*Pomatorhinus*	*Pomatorhinus ruficollis*
Passeriformes	Turdidae	*Geokichla*	*Geokichla citrina*
Passeriformes	Turdidae	*Geokichla*	*Geokichla sibirica*
Passeriformes	Turdidae	*Turdus*	*Turdus cardis*
Passeriformes	Turdidae	*Turdus*	*Turdus eunomus*
Passeriformes	Turdidae	*Turdus*	*Turdus hortulorum*
Passeriformes	Turdidae	*Turdus*	*Turdus mandarinus*
Passeriformes	Turdidae	*Turdus*	*Turdus obscurus*
Passeriformes	Turdidae	*Turdus*	*Turdus pallidus*
Passeriformes	Turdidae	*Zoothera*	*Zoothera aurea*
Passeriformes	Zosteropidae	*Staphida*	*Staphida torqueola*
Passeriformes	Zosteropidae	*Zosterops*	*Zosterops simplex*
Piciformes	Picidae	*Blythipicus*	*Blythipicus pyrrhotis*
Piciformes	Picidae	*Picus*	*Picus canus*
Strigiformes	Strigidae	*Otus*	*Otus lettia*

Table S2. Mammalian species information at sampling sites monitored by infrared-triggered camera

**Order**	**Family**	**Genus**	**Species**
Cetartiodactyla	Bovidae	*Capricornis*	*Capricornis milneedwardsii*
Pholidota	Manidae	*Manis*	*Manis pentadactyla*
Cetartiodactyla	Cervidae	*Elaphodus*	*Elaphodus cephalophus*
Cetartiodactyla	Cervidae	*Muntiacus*	*Muntiacus crinifrons*
Eulipotyphla	Erinaceidae	*Erinaceus*	*Erinaceus amurensis*
Primates	Cercopithecidae	*Macaca*	*Macaca mulatta*
Carnivora	Mustelidae	*Mustela*	*Mustela sibirica*
Rodentia	Sciuridae	*Callosciurus*	*Callosciurus erythraeus*
Rodentia	Hystricidae	*Hystrix*	*Hystrix brachyura*
Rodentia	Sciuridae	*Tamiops*	*Tamiops swinhoei*
Carnivora	Felidae	*Prionailurus*	*Prionailurus bengalensis*
Carnivora	Mustelidae	*Mustela*	*Mustela kathiah*
Lagomorpha	Leporidae	*Lepus*	*Lepus sinensis*
Carnivora	Mustelidae	*Melogale*	*Melogale moschata*
Carnivora	Mustelidae	*Arctonyx*	*Arctonyx collaris*
Carnivora	Prionodontidae	*Paguma*	*Paguma larvata*
Cetartiodactyla	Suidae	*Sus*	*Sus scrofa*
Rodentia	Sciuridae	*Dremomys*	*Dremomys pernyi*
Cetartiodactyla	Cervidae	*Muntiacus*	*Muntiacus reevesi*

Table S3. Number of bird and mammal studies under different stand types

Forest Types	Stand Type	Camera Number		Species Number		Independently Effective Record		Average Photos Per Camera	
		Bird	Mammal	Bird	Mammal	Bird	Mammal	Bird	Mammal
Secondary Forest	Broad-leaved	58	58	68	17	8586	11802	148	203
	Mountain Shrub	21	21	52	14	4622	5946	220	283
Plantation Forests	Moso Bamboo	51	61	45	15	1852	4896	36	80
	Cunninghamia lanceolata	40	43	51	15	2028	4855	51	113
Total	4	170	183	72	19	17088	27499	114	170

Figure S1. Habitat information of birds and mammals

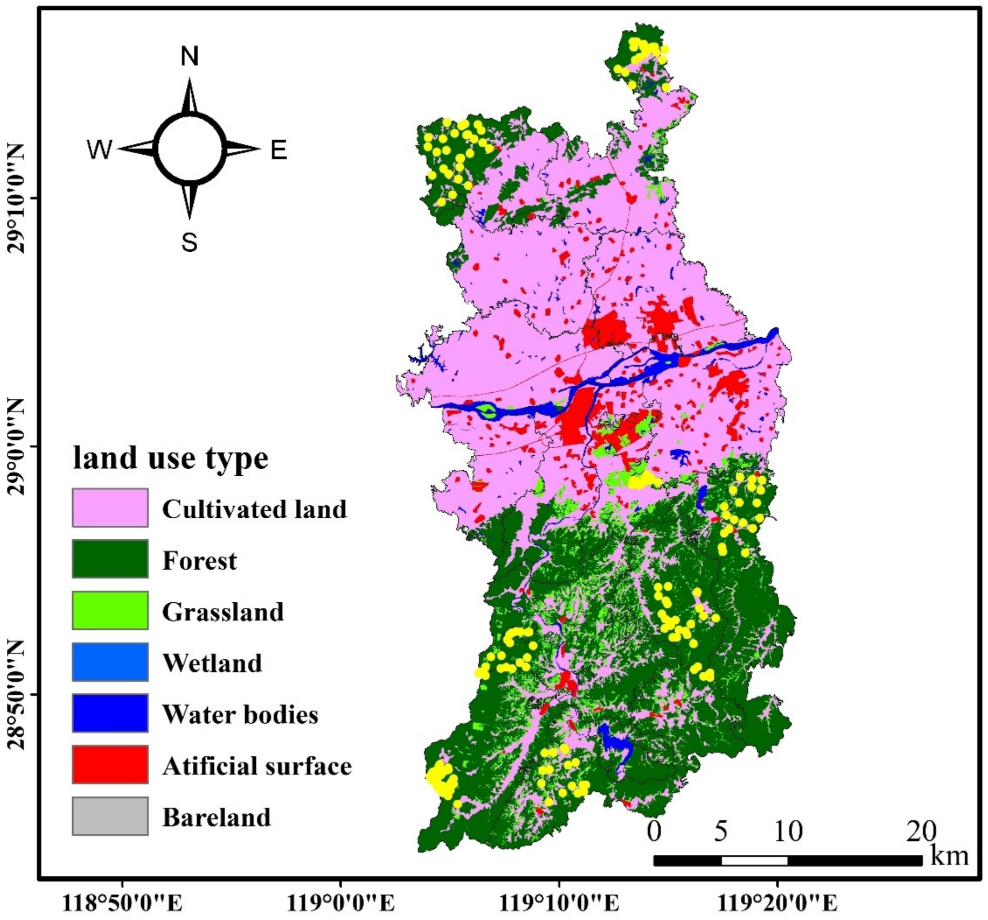

Figure S1. Distribution map of 203 infrared-triggered camera sites in Longyou County. yellow circles represent camera sites

Figure S2. Bird and mammal diversity and community structure between secondary and plantation forests

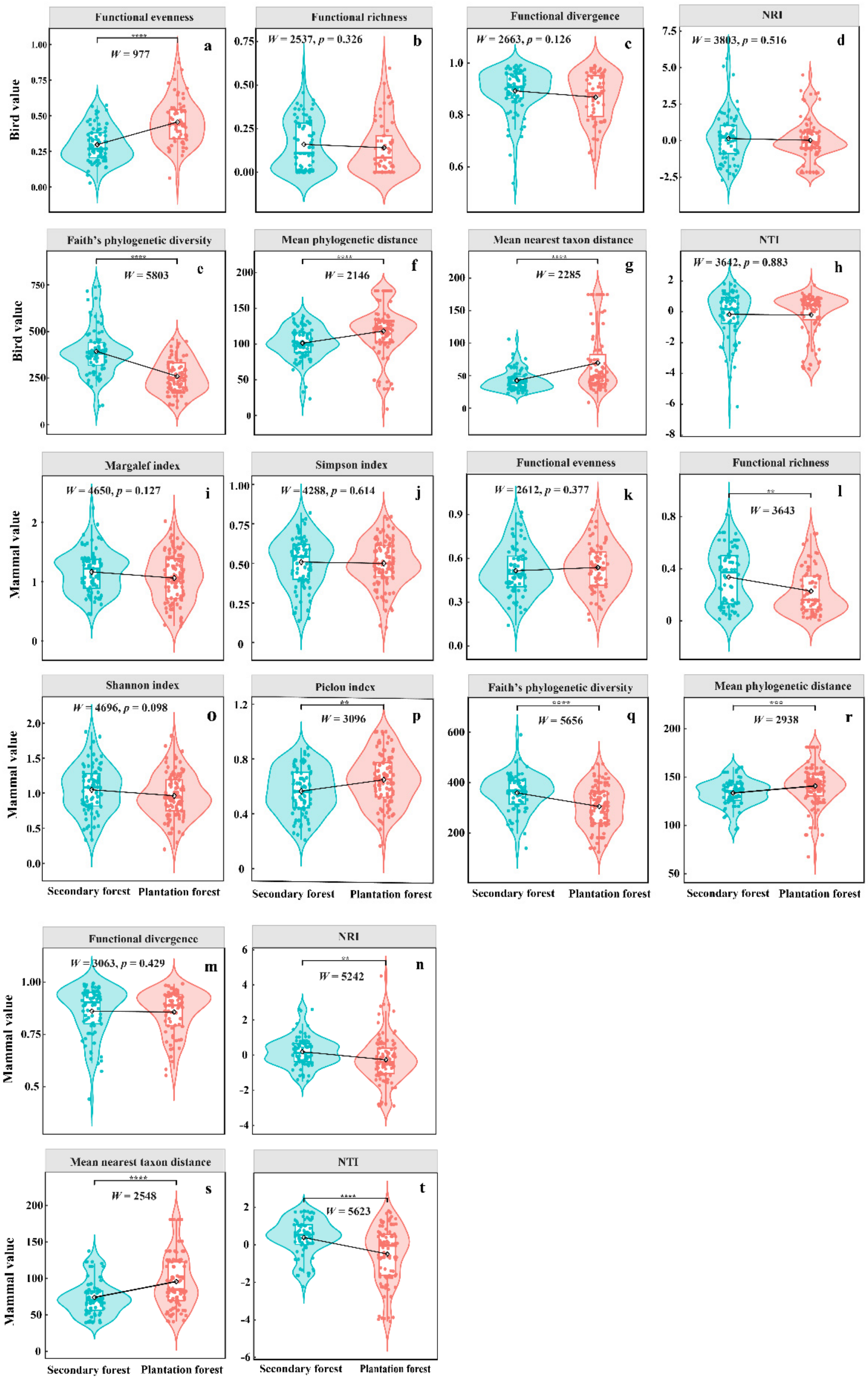

Figure S2. The differences of bird and mammal diversity and community structure between secondary and plantation forests based on Mann-Whitney U test. For birds, the group samples of different forests are from 60 to 91; For mammals, the group samples of different forests are from 75 to 104. The diamond symbols represent the mean value of the specific metric. ** $p < 0.01$, *** $p < 0.001$,**** $p < 0.0001$.
